# Single Crystalline Film of Hexagonal Boron Nitride Atomic Monolayer by Controlling Nucleation Seeds and Domains

**DOI:** 10.1038/srep16159

**Published:** 2015-11-05

**Authors:** Qinke Wu, Ji-Hoon Park, Sangwoo Park, Seong Jun Jung, Hwansoo Suh, Noejung Park, Winadda Wongwiriyapan, Sungjoo Lee, Young Hee Lee, Young Jae Song

**Affiliations:** 1 SKKU Advanced Institute of Nanotechnology (SAINT), Sungkyunkwan University (SKKU), Suwon, 440-746, Korea; 2 Center for Integrated Nanostructure Physics, Institute for Basic Science (IBS), Sungkyunkwan University (SKKU), Suwon, 440-746, Korea; 3 Nano Electronics Lab., Samsung Advanced Institute of Technology (SAIT), Suwon, 443-803, Korea; 4 Interdisciplinary School of Green Energy and Low Dimensional Carbon Materials Center, Ulsan National Institute of Science and Technology (UNIST), Ulsan, 689-798, Korea; 5 Center for Human Interface Nanotechnology (HINT), Sungkyunkwan University (SKKU), Suwon, 440-746, Korea; 6 College of Information and Communication, Sungkyunkwan University (SKKU), Suwon, 440-746, Korea; 7 Department of Physics, Sungkyunkwan University (SKKU), Suwon, 440-746, Korea

## Abstract

A monolayer hexagonal boron nitride (h-BN) film with controllable domain morphology and domain size (varying from less than 1 μm to more than 100 μm) with uniform crystalline orientation was successfully synthesized by chemical vapor deposition (CVD). The key for this extremely large single crystalline domain size of a h-BN monolayer is a decrease in the density of nucleation seeds by increasing the hydrogen gas flow during the h-BN growth. Moreover, the well-defined shape of h-BN flakes can be selectively grown by controlling Cu-annealing time under argon atmosphere prior to h-BN growth, which provides the h-BN shape varies in triangular, trapezoidal, hexagonal and complex shapes. The uniform crystalline orientation of h-BN from different nucleation seeds can be easily confirmed by polarized optical microscopy (POM) with a liquid crystal coating. Furthermore, seamlessly merged h-BN flakes without structural domain boundaries were evidence by a selective hydrogen etching after a full coverage of a h-BN film was achieved. This seamless large-area and atomic monolayer of single crystalline h-BN film can offer as an ideal and practical template of graphene-based devices or alternative two-dimensional materials for industrial applications with scalability.

Hexagonal boron nitride (h-BN), which is also called “white graphene”, has been paid special attention for its various outstanding properties including high thermal conductivity^1^, a low dielectric constant^2^, chemical inertness^3^,^4^, and high mechanical strength^1^. The h-BN, therefore, has a wide range of applications from protective coating, thermal interface material, transparent membrane and deep UV optoelectronic devices^1^,^2^,^3^,^4^,^5^,^6^,^7^,^8^,^9^,^10^. Furthermore, due to its very small lattice mismatch (~2%) with graphene, atomic-scale smoothness and free of dangling bonds, the h-BN has been regarded as the perfect substrate for graphene device applications. The h-BN exfoliated from bulk h-BN has been reported previously that it can be used as a substrate for graphene device instead of SiO_2_^5^, resulting in the graphene mobility improvement by an order of magnitude. Reasonably, the high-quality CVD-grown h-BN with large domain size can improve the performance of CVD-grown graphene device as well^9^,^11^. Thus, combining the high quality CVD-grown h-BN and graphene is a promising method to enable the commercial applications of high-performance graphene devices, such as capacitors and field-effect tunneling transistors^12^,^13^. Therefore, synthesis of the high-quality and large-area h-BN film is stringently required.

Of all the methods to synthesize h-BN, the CVD is one of the most potential methods to achieve a large-area and high-quality h-BN film. Until now, efforts have been devoted to synthesis of h-BN by atmospheric pressure CVD (APCVD) and low pressure CVD (LPCVD) on various substrates such as Pt^14^,^15^, Ni^16^, Cu^9^,^11^,^17^,^18^,^19^ and Ru^20^. However, the number of h-BN layers varies from mono-layer^9^,^14^,^17^,^19^, bi-layer^15^ to multiple layers^11^,^16^,^18^,^21^, while their domain size is still limited to a few micrometers^9^,^15^,^17^,^19^ with the shape of triangle^9^,^17^,^19^, diamond^19^ or hexagon^17^. Distribution of h-BN domains on a Cu surface is mostly random with different crystalline orientations, forming a large amount of boundaries, resulted in scattering defect generation. A number of defects significantly deteriorate the h-BN quality such as mechanical strength and electrical leakage. Therefore, to guarantee the high quality of the h-BN film, the most effective approach is to maximize domain size of a h-BN film with less boundaries or even the seamless stitch between different domains, like the case of wafer-scale single crystal graphene grown on Ge substrate^22^.

## Results

Seamless h-BN monolayer film with a uniform crystalline orientation on a Cu foil was successfully synthesized by CVD. Millimeter size of atomic monolayer of h-BN can be achieved by reducing the density of nucleation seeds for a triangular shape of h-BN flakes and by controlling the crystalline orientations of h-BN flakes. A very large domain size of a single crystalline monolayer h-BN grown by our method shows two important growth features; (1) the density of nucleation seeds with well-defined flake shape can be controllably reduced and (2) most of h-BN flakes have the same crystallographic orientation with parallel edges between h-BN flakes within Cu domains. With these features, we could finally get a seamless atomic-monolayer of h-BN film on Cu in large scale, at least few millimeter size defined by a Cu domain size, similar to the growth of large scale single crystal graphene on Ge or Cu substrates^22^,^23^,^24^. As h-BN is optically very transparent over 97%, it is hard to observe it directly on substrates like Cu, Pt or SiO_2_ substrates^9^,^14^. Here, we used a simple and quick method to observe h-BN domains on Cu easily with optical microscopy (OM) by oxidization in air (see Supplementary Fig. S1).

## Discussions

**Figure 1:**
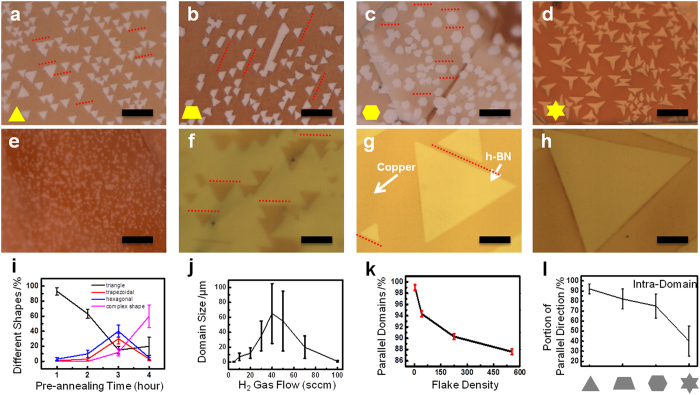
The control of domain shape and size of h-BN flakes. (**a**) to (**d**) are the OM images of h-BN domain grown on Cu foil with Ar gas (30 sccm) pre-annealing time varying from 1 hour, 2 hours, 3 hours and 4 hours, and then grow under H_2_ gas (10 sccm) and show different kinds of h-BN domain shapes. (**e**) to (**h**) are the OM images of triangle-shape h-BN domain grown under different H_2_ gas flow, varying from 5 sccm, 20 sccm, 30 sccm and 40 sccm, after 1 hour of Ar gas pre-annealing. (**i**) is the diagram to show the change of the percentage of different shapes of h-BN varying with Ar (30 sccm) gas pre-annealing time; (**j**) is the diagram to show the change of domain size of triangle-shape h-BN varying with hydrogen gas flow; (**k**) is the diagram to show the percentage of parallel domains varying with flake density; (**l**) is the diagram to show the portion of parallel direction of different h-BN domain shapes. The counted h-BN domains show in diagram of (**i**,**k**,**l**) are within the area of 60 μm × 50 μm; All the scale bars in (**a**–**g**) are 10 μm, and in (**h**) is 20 μm.

Figure 1 shows the first growth feature of controlling the shape of h-BN flakes and the domain size of the nucleation seeds. The dominant shape of h-BN flakes (yellow diagrams in each figure) could be selectively controlled by the pre-annealing time of a bare Cu foil before the h-BN growth as shown in Fig. 1a–d for 1, 2, 3 and 4 hours of pre-annealing under Ar (30 sccm) gas, respectively^17^,^25^. Careful observations show that the parallel alignment of h-BN flakes also get worse with longer pre-annealing time. While the pre-annealing condition of Fig. 1a is applied to keep a simple shape with better parallel alignments, different H_2_ flows of 5, 10, 20, 30 and 40 sccm are used. Figure 1e–h show that the domain size of single crystal h-BN increases from less than 1 μm to more than 100 μm with H_2_ gas flow increasing. All the effects on the dominant shape of h-BN flakes and the domain size of nucleation seeds are statistically plotted in Fig. 1i–l. Figure 1i plots portions of typical h-BN flake shapes with different pre-annealing time of a bare Cu foil. The mechanism of the h-BN shape dependence on annealing time, however, has not been clear yet. The reason we guess is because since pre-annealing was done under Ar atmosphere without H_2_ at high temperature (1050 °C), the residual oxygen may exist in the CVD system. We assume that the concentration of oxygen would increase with annealing time and affect the Cu surface morphology and oxygen content, resulting in different shapes of h-BN crystals. In the previous study^17^, oxygen content plays important role to alter h-BN shape from triangle shape to hexagonal shape. The pre-annealing time of an initial Cu foil with one hour, therefore, is preferred in our work to selectively maintain the triangular shape of h-BN flakes. Figure 1j is the statistical data for the domain size of nucleation seeds with different H_2_ flows. The domain size increases dramatically by increasing the H_2_ flow up to an optimum flow rate of H_2_ gas (≤50 sccm) when the density of h-BN nucleation seeds is suppressed as discussed before. The h-BN growth itself, however, is suppressed with even higher H_2_ gas flow. Finally, h-BN can be hardly grown with over 100 sccm of H_2_ gas (see Supplementary Fig. S5). In addition, careful observations for Fig. 1e–h lead to find that most of h-BN flakes are parallel edges between h-BN flakes (the parallel red dashed lines in the figure). Furthermore, the h-BN domains are also observed to be parallel even across the Cu domain boundaries (see Supplementary Fig. S2). The portion of parallel alignments of h-BN flakes also depends on the density of the nucleation seeds. Figure 1k plots the portion of the parallel h-BN flakes for different density of nucleation seeds. When the density is as low as 0.01 μm^−2^, almost 98% of h-BN flakes are well aligned in the same direction. A larger size with better alignments for h-BN flakes can be achieved by appropriate pre-annealing time and H_2_ gas flow, and which suggest a way to grow the larger-area and atomic-monolayer of h-BN with single crystallinity, by seamlessly-merging between parallel h-BN flakes.

**Figure 2:**
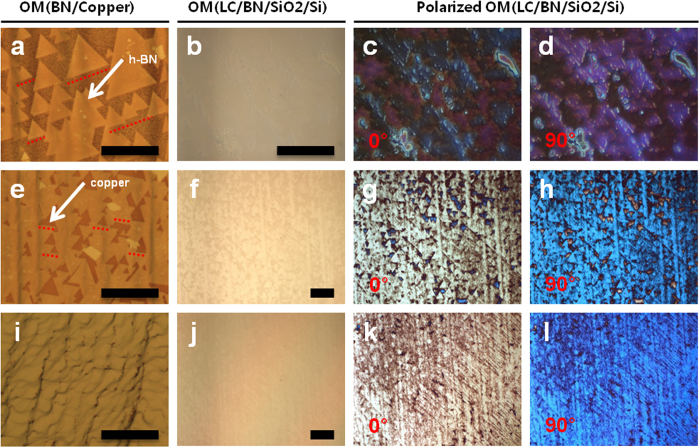
Polarized OM measurements to confirm the seamless stitch of h-BN flakes when merged together. (**a**,**e**,**i**) are the OM images of triangle-shape h-BN grown under hydrogen (40 sccm) gas with different growth time of 3 hours, 4 hours and 5 hours, respectively; (**b**,**f**,**j**) are the corresponding h-BN from (**a**,**e**,**i**), after transferring onto SiO_2_ substrate and with LC coating on; (**c**,**g**,**k**) are the polarized OM images with the angle of 0°, which are the same part in (**b**,**f**,**j**); (**d**,**h**) and (**l**) are the polarized OM images with the angle of 90°, which are the same part in (**b**,**f**,**j**); All the scale bar are 100 μm.

Longer growth time is used to achieve a full coverage of single crystalline and atomic-monolayer of h-BN with the help of the previously described two growth features. Then, each h-BN flake on the surface gets merged together making a full one monolayer coverage. Figure 2a,e,i show the different coverages of h-BN at growth times of 3, 4 and 5 hours, while keeping the same conditions of pre-annealing time of 1 hour and H_2_ gas flow of 40 sccm to reduce density of nucleation seeds with a triangular shape of h-BN flakes. Figure 2a clearly shows both of the growth features (the reduced density of nucleation seeds and the alignment between h-BN flakes). In a very few cases, a small size of bilayer h-BN flakes can be also observed under the middle of some large h-BN flakes while keeping the same shape and alignment with its mother layer. When grown with longer growth time of 4 hours, the h-BN covers more, and the exposed Cu surface still keeps triangular shapes because of growth dynamics (Fig. 2e). Finally, one full monolayer of h-BN is achieved at 5 hours of h-BN growth as shown in Fig. 2i. The nematic liquid crystal (LC)-assisted polarized optical microscopy (POM) is a very simple and rapid method to directly observe the size and orientation of the graphene and h-BN flake by using the self-alignment of LC^26^. Figure 2b,f,j show OM images of h-BN on SiO_2_ substrate (285 nm thick) coated with the nematic LC molecules (5CB, E. Merck). The h-BN flakes are hardly observed. Figure 2c,g,k and Fig. 2d,h,i show the POM images on rotational of the sample for 0° and 90°, respectively. The colors of each h-BN flake were similarly changed for the rotation angles of 0° and 90° in the POM images indicating that the h-BN has a similar orientation within the flakes. Larger domain size of single crystalline h-BN monolayer was confirmed by POM measurements with LC coating as shown in Fig. 2c,d,g,h,k,l.

**Figure 3:**
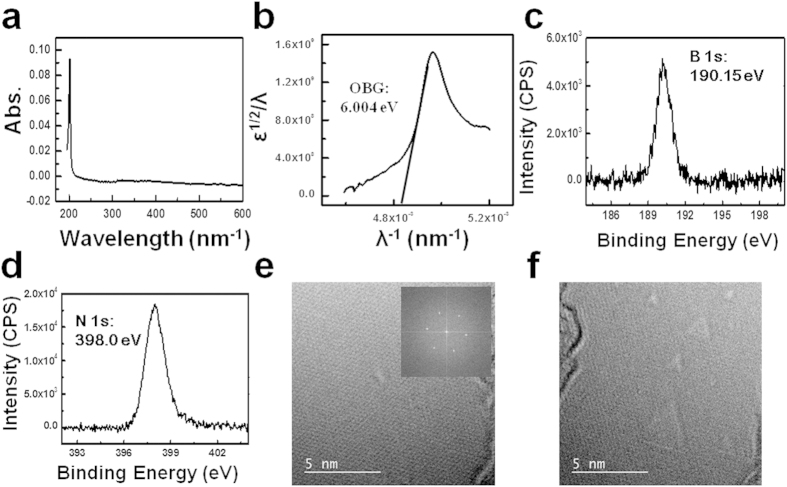
Characterizations by UV, XPS, and TEM. (**a**) is the UV-visable absorption spectrum, transferred onto quartz substrate; (**b**) is the corresponding plot of ε^1/2^/λ versus 1/λ of (**a**); (**c**,**d**) are the B1s spectrum and N1s spectrum, respectively; (**e**) is the HR-TEM image of the h-BN film, after transferring onto Si_3_N_4_ grid and the inset image is the corresponding FFT image; (**f**) is the HR-TEM image of h-BN film after exposing under the electron beam for 20 seconds, showing some parallel triangle holes.

To confirm the quality of our one full monolayer of h-BN, it was transferred onto quartz substrate. Figure 3a shows its UV-visible spectrum. Obviously, the h-BN peak at 201 nm is very sharp. Figure 3b shows its analysis which implies that the band gap of this h-BN film is 6.004 eV proving its high quality^18^. Next, the X-ray photoemission spectroscopy (XPS) measurement was also done on the h-BN/Cu sample. XPS spectra of B1s and N1s are shown in Fig. 3c,d, respectively. To confirm the crystallinity of the synthesized one full monolayer of h-BN in an atomic scale, high resolution transmission electron microscopy (HR-TEM) was measured as shown in Fig. 3e, which shows a perfect atomic structure of the monolayer h-BN. The inset image is the fast Fourier transformed data (FFT), where 6-fold peaks are clearly seen. To intentionally generate artificial damages to the h-BN monolayer, focused electron beam (e-beam) was exposed to the sample for ~20 seconds. Figure 3f shows that intentional etching by focused e-beam generates triangular holes in it, when there is no domain boundary. Additionally, the transferred h-BN domains with triangle, trapezoidal and hexagonal shapes on SiO_2_ substrates were measured by atomic force microscopy (AFM). The h-BN thickness is approximately 0.78 nm (see Supplementary Fig. S3), which indicates uniform monolayer.

POM with LC coating in Fig. 2 reveals that a large-area and atomic monolayer of h-BN could be formed with a single crystallinity by applying the two growth features. To further confirm if the h-BN flakes are seamlessly merged without any structural boundaries, H_2_ etching technique was utilized as its simple and quick method. H_2_ can selectively etch out the point defects or the domain boundaries as previously studied for the CVD graphene on Cu^20^,^27^,^28^. The previous researches reported that the H_2_ etching starts from energetically unstable parts such as point defects or edges of a single crystalline graphene flake. Then H_2_ etching propagates along domain boundaries, or makes epitaxial-etching with triangular/hexagonal holes. In our work, this method was applied to single-crystalline and poly-crystalline h-BN flakes to confirm if the selective H_2_ etching can identify domain boundaries. As shown in Fig. 1, the shape of h-BN flakes can be controlled by pre-annealing time before h-BN growth. When an atomic monolayer of h-BN is fully grown, the H_2_ gas flow of 20 sccm was introduced without any h-BN precursor while keeping the same growth temperature of 1050°C.

**Figure 4:**
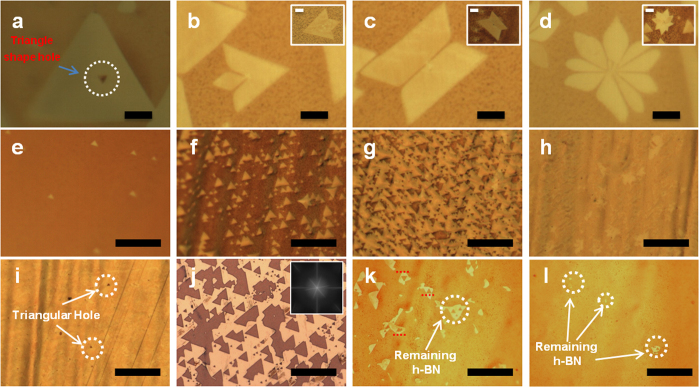
Selective hydrogen etching to reveal the defects and boundaries of h-BN. (**a**) is the OM image of h-BN domain with a triangle-shape hole in, after hydrogen etching; (**b**) to (**d**) are the OM images of complex-shape of h-BN domains with gap between neighboring parts, after hydrogen (20 sccm) etching, the inset images show the complex-shape h-BN domains have complete shape before hydrogen etching; (**e**) to (**h**) are the OM images of triangle-shape h-BN domain growth under 20 hydrogen gas flow with different growth time, varying from 10 minutes, 20 minutes, 40 minutes to 60 minutes; (**i**) to (**l**) are the OM images that show hydrogen etching on the complete fully covered h-BN film from (**h**) with different etching time of 5 minutes, 20 minutes, 50 minutes and 80 minutes, and the inset image in (**j**) is the corresponding FFT image. The scale bars in (**a**) to (**d**) are 10 μm and in (**e**) to (**l**) are 40 μm.

Figure 4a shows that the simple shape (triangle) of a h-BN flake keeps its original shape, however, sometimes with a small triangular hole in it. This original h-BN flake was grown on a Cu foil, which was pre-annealed for 1 hour. This hole is believed to come from an epitaxial etching out of a point defect as reported for graphene^27^,^28^. The different h-BN flakes in Fig. 4b–d, however, were grown on a Cu foil, which was pre-annealed for 3 hours. These h-BN flakes show some complicate shapes with etched lines in them after the selective H_2_ etching. Each inset image is the corresponding h-BN flakes before the H_2_ etching. The complicated shapes of h-BN flakes show a very interesting feature. Possible boundaries with different crystalline orientations are etched out as lines. We believe that h-BN flakes in Fig. 4b–d are all polycrystalline with domain boundaries, and those domain boundaries are energetically unstable and easy to be etched out. The flakes in Fig. 4b–d (after hydrogen etching) are not the same flakes with each corresponding inset image (before hydrogen etching). As it was hardly possible to find out the exactly same flake before and after the hydrogen etching, we had to compare many flakes of similar shapes for hydrogen etching effect, which show statistically the same conclusion. By using this selective H_2_-etching of domain boundaries, domain sizes or domain boundaries in a h-BN monolayer can be easily and quickly investigated in large scale. Figure 4e–h shows different coverages of h-BN with different growth times of 10, 20, 40 and 60 min, respectively, under H_2_ gas flow of 20 sccm. The h-BN flakes are growing in the shape of parallel triangles as growth time increases to form a full coverage of a h-BN monolayer. Figure 4i–l are OM images showing the H_2_-etching of a h-BN monolayer with different etching times of 5, 20, 50 and 80 min, respectively. All the etched parts are epitaxial triangular holes and propagate having the same edge orientations (as shown by the red dashed line) as a mother layer without any etched line. The inset in the Fig. 4j is the corresponding FFT result from the Fig. 4j. Interestingly, this inset shows the perfect six-fold symmetry (3 fold times 2), indicating that all the edges of etched h-BN are parallel each other. This implies that this large-area and atomic monolayer of h-BN is single crystalline without structural domain boundaries^28^. All the Cu foils used for Fig. 4e–l were pre-annealed for 1 hour.

**Figure 5:**
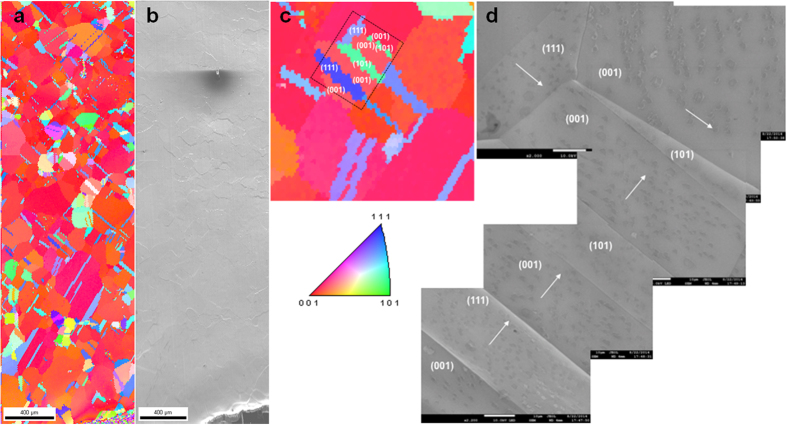
EBSD measurements to compare the orientation of h-BN flakes and the facet of the underlying Cu. (**a**) is the EBSD data of large area of Cu (with 2 hours of Ar pre-annealing) surface with short time annealing (Ar/H_2_), after h-BN grown on; (**b**) is the corresponding SEM data of (**a**); (**c**) is the EBSD image of small size Cu foil with many domains having different crystal orientations on; (**d**) is the corresponding SEM image of (**c**).

To understand the correlation of the orientation of h-BN flakes and the crystal direction of the underneath Cu domain, the electron back-scattering diffraction (EBSD) was performed. Figure 5a,b show the EBSD and SEM data at the same position. The different colors in EBSD data indicate different crystal orientations of the underlying Cu. The dominant plane of the Cu foil used in this work was found to be Cu(001). Figure 5c is a magnified EBSD data including many different crystal facets, and Fig. 5d is a series of SEM images on the corresponding locations marked with crystal facets (coordinates) and h-BN direction (arrow). Although all the flakes in any single Cu domain are parallel, any correlation between the crystal facets of Cu and the orientation of h-BN flakes has not been found unlike the previously reported case of large-area graphene^23^,^24^. The scale bar in Fig. 5a is 400 μm, and dominant sizes of Cu domains are large enough around 100’s μm, of course, with many small domains distributed randomly over the surface. Unlike the previous experiments and calculations^29^,^30^ our observations by OM, SEM and EBSD show the orientation of h-BN flakes has no correlation with the crystal facets of the underlying Cu foil. And interestingly, because of this important feature, large-area h-BN monolayer can be grown over different Cu domain boundaries with keeping the same orientations. If the potential barrier by atomic structure of Cu surfaces is critical, then large-area h-BN monolayer could not be grown. We also performed DFT calculations with various sizes of flakes on Cu(100) and (111) surfaces, and we arrived at the conclusion that the alignment in the h-BN triangles cannot be attributed to the intrinsic interaction between h-BN flakes and the Cu surface. The predominant appearance of the triangular shape suggests that the edges terminate with a single species, either B or N. Since B-terminated edges are much more unstable, due to its dangling bond nature, and thus we thought that the triangles are more likely terminated with N atoms. However, we could not find any mechanism that determines the flake direction on a Cu surface. For example, the Cu(100) is the 2D square lattice with bond lengths of 2.553 angstroms. The strong bond formation between the h-BN edges and the Cu surface can hardly fix a particular orientation of micron-scale h-BN flakes. On the other hand, without the B-terminated dangling bond edges, the h-BN flakes are floating on Cu surface through the van der Waals interaction, in which case the h-BN orientation does not depend on orientation of facet of Cu surface. The dominant appearance of triangle shapes and the orientational alignment within the h-BN flakes seems to be determined by conditions of growth situation, and we concede that we cannot find any clue for it in the surface interaction between h-BN and Cu.

In conclusion, we successfully synthesized the large-area and atomic monolayer of h-BN on Cu up to the maximum sizes of ~mm defined by a Cu domain size by LPCVD. This high quality h-BN monolayer can be achieved by controlling two growth features. The first one is the reduced density of the nucleation seeds with a well-defined flake shape, by pre-annealing of a bare Cu foil before h-BN growth and H_2_ effect during the growth. Single crystalline h-BN flakes can be grown up to 50 ~ 100 μm. By this method, a chance to have a defective boundary can be avoided. The second feature is the alignment of h-BN flakes to make h-BN flakes merged seamlessly within each Cu domain without structural domain boundaries^28^. The origin for the alignment of h-BN flakes is not clear yet, however, the single crystallinity of the full layer was confirmed by POM and selective H_2_ etching. With further optimization for a Cu foil, therefore, this study on large-area and atomic-monolayer of single crystalline h-BN can offer an ideal template of graphene for industrial applications with scalability^6^.

## Methods

### Cu foil pre-treatment

A Cu foil from Alfa Aesar (no. 13382) was used for growth substrate. The Cu foil was then chemically polished in a solution containing 20 g potassium persulfate, 98%, and 500 mL DI water under an applied current of 0.6 A for 10 min to remove impurities and the native oxide. By this method, the impurities or native oxide on surface can be removed. Secondly, the Cu was rinsed by HF (20%) for 3 times to remove etchant solvent residue. Finally, the Cu was dried by a N_2_ gun.

### Growth details

To grow single crystal h-BN, firstly the Cu foil was annealed at 1050 °C for a designated time, according to a desirable h-BN shape. After annealing, the borazine was introduced to the chamber by the carrier gas N_2_ with a gas flow of 0.5 sccm. Meanwhile, H_2_ and Ar gases were used to dilute the borazine, with a gas flow of 100 sccm and 70 sccm, respectively. After 30 min growth, the carrier gas N_2_ was turned off to stop h-BN growth. A small gas flow of H_2_ was continuously flowed during the cooling process for the H_2_ etching of h-BN.

### Transfer

To transfer the h-BN grown on Cu to other substrates, like SiO_2_/Si, quartz, and TEM grid, firstly, the sample was spin-coated with PMMA, and then the sample on the backside of the Cu foil was removed by plasma etching. Next, the sample with PMMA coating was put into the Cu etchant (an iron (III) chloride solution, concentration of 0.1 g mL^−1^) for over 12 hours. The h-BN/PMMA film was then rinsed in DI water twice and then in the HCl (concentration of 0.3 g mL^−1^) for 10 minutes to remove the residual iron (III) chloride, and at last use the DI water to rinse the sample again. Finally, the sample was transferred onto the target substrates; quartz, SiO_2_ substrate or Si_3_N_4_ TEM grid. After the sample was dry, the PMMA was then subsequently removed by heated acetone (on hot plate with 110 °C) for 3 minutes.

### Characterization

X-ray photoelectron spectroscopy (XPS) was carried out on an ESCA Lab2201-XL spectrometer using an Al Kα X-rays as the excitation source. The morphology and crystal characterization were conducted with a transmission electron microscope (TEM 2100F, JEOL) equipped with selected area diffraction (SAED). Atomic force microscopy images were acquired in air cantilevers operated in tapping mode.

## Additional Information

**How to cite this article**: Wu, Q. *et al.* Single Crystalline Film of Hexagonal Boron Nitride Atomic Monolayer by Controlling Nucleation Seeds and Domains. *Sci. Rep.* **5**, 16159; doi: 10.1038/srep16159 (2015).

## Supplementary Material

Supporting Information
