# Supplementary material for: Single Crystalline Film of Hexagonal Boron Nitride Atomic Monolayer by Controlling Nucleation Seeds and Domains
Source: Sci Rep. 2015 Nov 5;5:16159. doi: 10.1038/srep16159 (PMC4633619; doi:10.1038/srep16159)
Supplement: Supporting Information [file srep16159-s1.pdf]

Supporting information for

Single Crystalline Film of Hexagonal Boron Nitride Atomic Monolayer by Controlling Nucleation Seeds and Domains

Qinke Wu[1], Ji-Hoon Park[2], Sangwoo Park[1], Seong Jun Jung[1], Hwansoo Suh[3], Noejung Park[4], Winadda Wongwiriyapan[1,5], Sungjoo Lee[1,5,6,], Young Hee Lee[2,*], and Young Jae Song[1,2,7,*]*

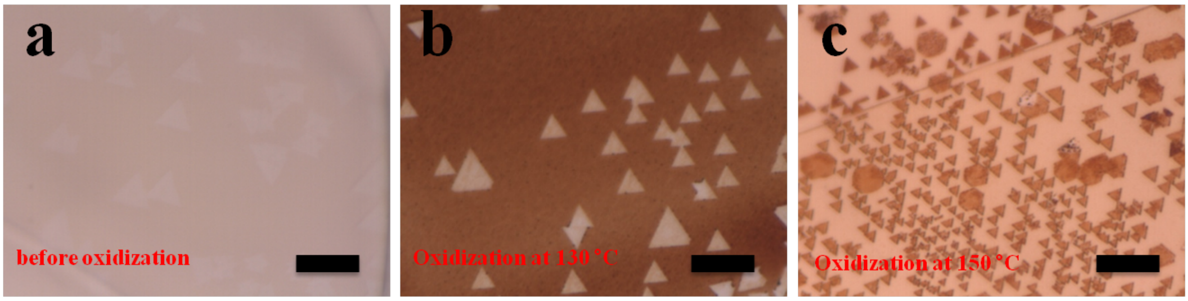

Figure S1: Clear OM observation of h-BN flakes by Cu oxidation. (a) is the optical microscopy image of the original Cu foil with h-BN on it, without oxidization; (b) is the optical microscopy image of the Cu foil with h-BN on it, after oxidization at 130 °C for 7 hours; (c) is the optical microscopy image of the Cu foil with h-BN on it, after oxidization at 150 °C for 7 hours. All the scale bars are 10 µm.

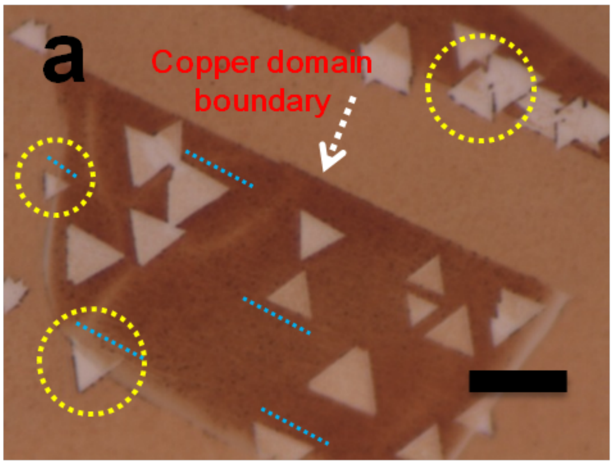

Figure S2: Growth across the Cu boundary. (a) is the OM image which shows the h-BN domains (in the dashed yellow circle), which grow across the Cu boundary, even keeps the same crystalline orientation with the ones within Cu domains. The scale bar is 10 μm.

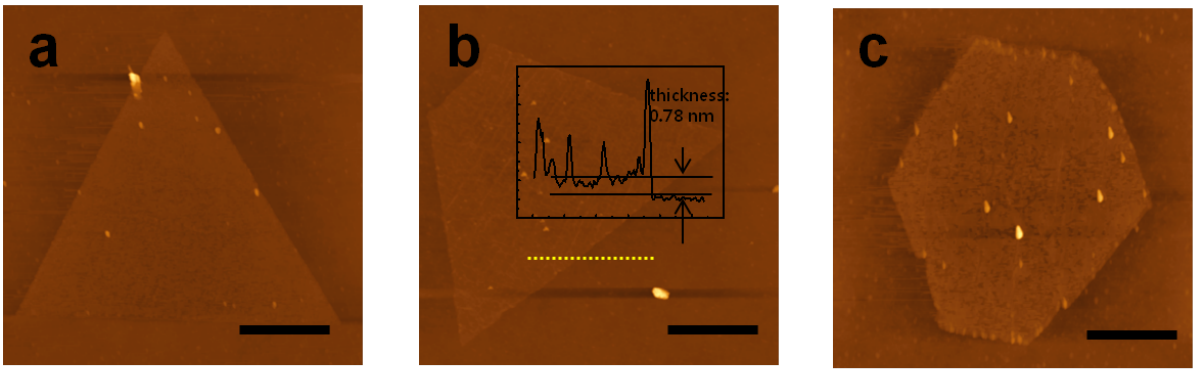

Figure S3: (a) to (c) are the atomic force microscopy (AFM) images of triangle shape, trapezoidal shape and hexagonal shape of h-BN domains transferred onto the SiO_2 substrate, respectively; the inset in (b) image is the diagram along the dashed-yellow line, which shows the monolayer h-BN has a thickness of around 0.78 nm and it matches well with previous results. All the scale bars are 2 μm.

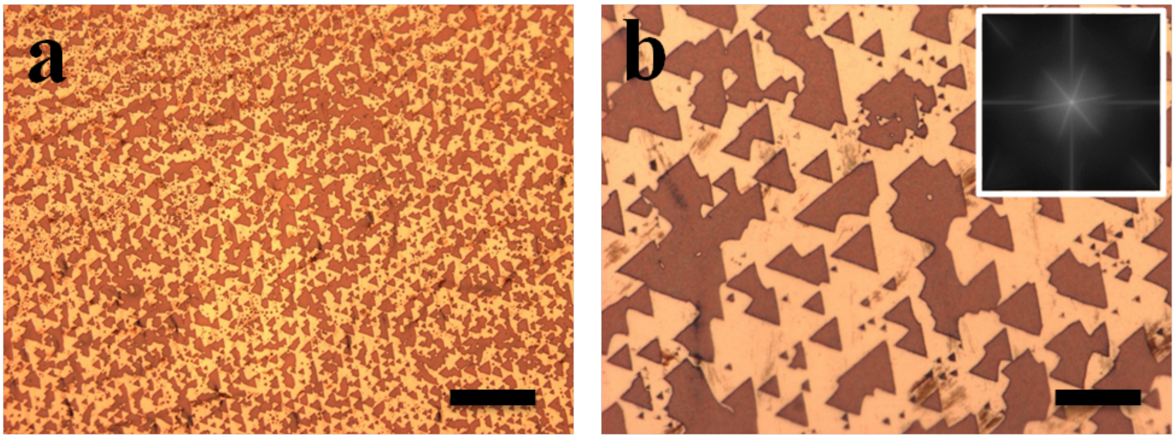

Figure S4: (a) is the large area of h-BN with parallel triangle holes, after hydrogen etching; (b) is the zoomed in OM image, and the inset image is the corresponding FFT image. The scale bars in (a) is 150 µm, in (b) is 20 µm.

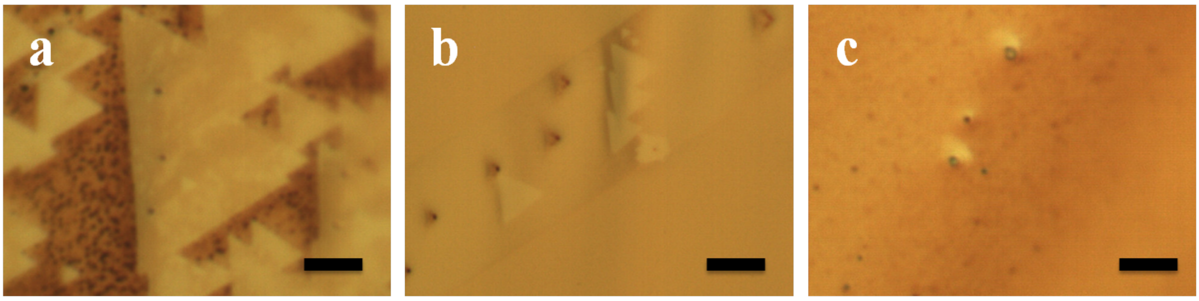

Figure S5: (a) is the OM image of h-BN growth under 55 sccm; (b) is the OM image of h-BN growth under 70 sccm; and (c) is the OM image of h-BN growth under 100 sccm. The scale bare in (a) to (c) are 50 µm, 50 um and 20 µm, respectively.
